# Implementation of Isoniazid Preventive Therapy Among HIV-Infected Children at Health Facilities in Nairobi County, Kenya: A Cross-Sectional Study

**DOI:** 10.24248/EAHRJ-D-19-00005

**Published:** 2019-11-29

**Authors:** Peninah M Mwangi, Dalton Wamalwa, Diana Marangu, Elizabeth M Obimbo, Murima Ng’ang’a

**Affiliations:** a Department of Paediatrics and Child Health, University of Nairobi, Nairobi, Kenya; b Ministry of Health, Nairobi, Kenya

## Abstract

**Background:**

HIV is the strongest risk factor for developing tuberculosis (TB) among people with latent or new *Mycobacterium tuberculosis* infection. Isoniazid preventive therapy (IPT) reduces the risk of active TB among people living with HIV by up to 62%. Despite evidence that IPT is safe and efficacious, its provision remains low globally. The current study aimed at documenting IPT uptake, adherence, and completion rates, as well as the correlates of IPT uptake among HIV-infected children in Kenya. The study also assessed the knowledge, attitude, and practices of health-care workers (HCWs) with regard to IPT.

**Methods:**

A health facility-based cross-sectional study was conducted. Data were collected from caregivers of HIV-infected children as well as HCWs using an interviewer-administered questionnaire. Logistic regression was used to determine the factors associated with IPT uptake.

**Results:**

The study enrolled 111 child-caregiver dyads. Most of the caregivers were female (n=75, 77.3%) and HIV-positive (n=82, 85.4%). The majority of children were male (n=65, 58.6%) and on ART (n=106, 95.5%). Overall, 59 children were on IPT (uptake of 53.2%, 95% confidence interval [CI], 43.9% to 62.4%). Out of the 25 children who had been on IPT for more than 6 months, 22 (88.0%) successfully completed the 6-month course of treatment. Further, 27 of the 34 children (78.4%) who were on IPT at the time of the study demonstrated satisfactory adherence to the therapy (no doses missed). The caregivers’ attributes that were associated with IPT uptake included having a secondary school education (adjusted odds ratio [aOR] 0.13; 95% CI, 0.03 to 0.67) and having been on IPT (aOR 27.50; 95% CI, 5.39 to 140.28). The characteristics of children that were significantly associated with IPT uptake were higher median baseline CD4 count (*P*=.007) and higher median current CD4 count (*P*=.024).

**Conclusion:**

The study demonstrated suboptimal IPT uptake but favourable adherence and treatment completion rates. There was almost universal awareness of IPT within the study sample. Furthermore, the majority of the HCWs had a favourable attitude towards IPT. However, the attendant IPT practices were inadequate, with majority of HCWs reporting that they had never initiated IPT, prescribed IPT within the last 12 months, or renewed an isoniazid prescription.

## INTRODUCTION

The global burden of the HIV/AIDS is disproportionately concentrated in sub-Saharan Africa; 71% of people living with HIV reside in the region while 75% of deaths related to the HIV epidemic and 65% of new HIV infections occur in sub-Saharan Africa.^1,2^ Furthermore, morbidity and mortality from tuberculosis (TB) associated with HIV is a principal concern in sub-Saharan Africa. It is estimated that, in sub-Saharan Africa, 1 in every 3 TB cases is HIV-positive, with remarkable heterogeneity of the burden being observed in the region: prevalences of 25.5%, 31.1%, 41.3%, and 43.7% in the western, eastern, central, and southern regions of sub-Saharan Africa, respectively.^3^

Tuberculosis (TB) remains a major global health problem with annual estimates being 9 million new cases, 1.4 million deaths due to TB in addition to 430,000 Human immunodeficiency virus (HIV) associated TB deaths. The strongest risk factor for developing TB disease in those with latent or new *Mycobacterium tuberculosis* infection is HIV.^4^ The risk of developing TB is 21-34 times greater in people living with HIV than among those who do not have HIV infection.^5^ The African region accounts for about 4 out of every 5 TB-and-HIV-coinfected cases. Kenya ranks among the 22 high burden countries in the world and among the top 5 from sub-Saharan Africa.^6^ In 2013, Kenya reported a total of 89,796 of all forms of TB; paediatric age group less than 15 years constituted 6% of these.^6,7^ The annual incidence of TB among children in Kenya is 283/100,000.^7^

Isoniazid preventive therapy (IPT) is the use of the drug isoniazid in HIV-positive individuals with latent TB infection and under-5 contacts of PTB index cases in order to prevent the development of active TB disease. Studies have shown that IPT dramatically reduces the incidence of TB among people living with HIV.^8–11^ A 2004 Cochrane Review found that IPT reduced the risk of TB by 33% overall and by 64% when targeted to people living with HIV who had a positive tuber-culin skin test.^12^ IPT significantly reduces the incidence of TB even among people living with HIV and receiving ART.^13^ In 1998, World Health Organization (WHO) and Joint United Nations Programme on HIV/AIDS (UNAIDS) issued a statement that recognised the effectiveness of IPT among people living with HIV and recommended its use as part of an essential care package for these patients.^14^ IPT targets infants of mothers with smear negative or positive PTB, children under 5 years of age as TB contacts, HIV-infected individuals, health-care workers (HCWs) who are HIV-positive and HIV-positive prison inmates. WHO recommends isoniazid (10 mg/kg/day) for 6 months to all children living with HIV who are more than 12 months of age and in whom active TB has been ruled out through symptom based screening.^15^

Although global initiatives such as the United States President's Emergency Plan for AIDS Relief and the Global Fund to Fight AIDS, Tuberculosis and Malaria have focused on scaling up ART, millions of people living with HIV remain eligible for and could benefit from prophylaxis against TB thorough IPT.^16,17^

Following the launch of The Kenya IPT Policy in 2011, the Ministry of Health recommends IPT for 6 months for all eligible people whom TB has been excluded using the intensive case finding tool. Further, it defines the following clients as being eligible for IPT: HIV-infected children less than 12 months who have had recent close contact with sputum-positive TB disease with no evidence of active TB; All persons living with GHIV above 12 months (including pregnant and breastfeeding women) who screen negative for active TB; all children below 5 years, irrespective of HIV status, who have recent close contact with sputum positive TB disease with no evidence of active disease; and prisoners who screen negative for active TB (irrespective of their HIV infection).^18^ The purpose of the current study was to determine the level of uptake of IPT among HIV-infected children in Kenya, adherence and factors associated with it. The evidence generated from this study could be utilised in strengthening provision of IPT, scaling up the services as well as updating the national policy and guidelines on IPT.

## METHODS

### Study Design and Study Site

The study was conducted in Nairobi County, Kenya (Figure) involved a cross-sectional survey of 3 health facilities, namely Langata Health Centre (LHC), Mbagathi District Hospital (MDH), and Kenyatta National hospital (KNH). Kenyatta National hospital (KNH), which is located in Kibra Constituency, is categorised as a Level VI health facility (the highest level in provision of preventive and curative services). The hospital has a catchment population of 4 million but being 1 of only 2 major national referral hospitals in Kenya it receives patients from all over the country. By the time of conducting the present research, 1996 children were on follow up in the comprehensive HIV care clinic (CCC), 68% of whom are on ART. Mbagathi District Hospital (MDH) is a Level V health facility situated in Dagoretti Constituency. The hospital's catchment populations constitutes mainly people from the nearby Kibera informal low-income settlements. At the time of the study, 320 children (1–15 years) were enrolled in the CCC with the majority being on ART (98%). Langata Health Centre (LHC) is situated in Langata Constituency which on southwestern side of Nairobi City. It caters for a catchment population of about 80,000 people majorly from Gatwikira, Kianda, Southlands and Soweto informal low-income settlements. The bed capacity in LHC is 20.

**FIGURE.**
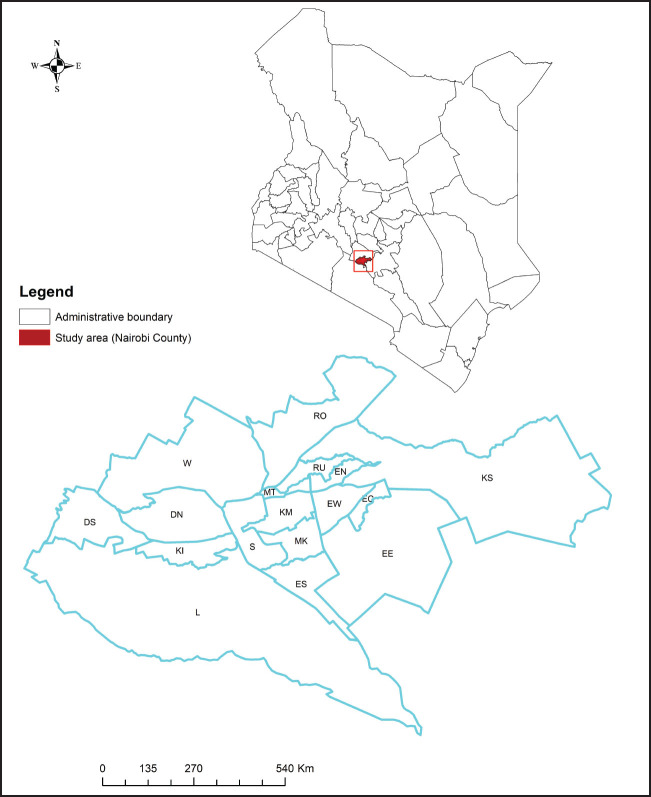
Map of the Study Area

### Study Population

The study population comprised of children enrolled in the CCC of KNH, MDH and LHC. Additionally, HCWs serving in the KNH, MDH and LHC were part of the study population. The inclusion criteria for the children included; having documented HIV infection, being in the age range of 1 to 15 years, and getting informed written consent from the caregiver. Children with active TB disease and those with contraindications to isoniazid (active hepatitis, peripheral neuropathy) were excluded. HCWs who were working in the CCC of KNH, MDH and LHC were included in the study while those who were not involved in administering IPT to children were excluded.

### Sample Size Determination

The minimum required sample size for the study was determined as described by Lwanga and Lemeshow.^19^

$$n_{0}=\frac{z_{a}^{2}p(1-p)}{d^{2}}$$

Where:

*n_0_*=minimum required sample size

*Z_a_*=value from the standard normal distribution corresponding to the 95% confidence level (1.962)

*P*=An estimate of the proportion of study participants with the characteristic of interest (0.5) d=desired degree of precision (0.1)

Thus,

$$n_{0}=\frac{1.962^{2}\times 0.5(1-0.5)}{0.1^{2}}=97\;\text{study}\;\text{subjects}.$$

Adjusting for a 10% nonresponse rate, the minimum required sample size for the study was 108 children.

For the HCWs, the sample size was adjusted for a finite population^20^:

$$n=\frac{n_{0}}{1+\frac{\left(n_{0}-1\right)}{N}}$$

Where *n* is the final minimum required sample size and N is the HCW population size (144).

Thus, $n=\frac{96.2}{1+\frac{96.2-1}{144}}=57.9$.

The minimum required HCW sample size was 58.

The samples were distributed among the health facilities using probability proportionate to population size technique.^21^

### Recruitment of Study Participants and Data Collection

The children and their caregivers were recruited consecutively as they sought services at the 3 health facilities. Each enrolled child was screened for TB using the WHO algorithm for TB screening.^22^ Based on the records obtained from the administrators of the study health facilities, random sampling approach was used to enrol the HCWs in the research. Data were collected from the caregivers and HCWs using an interviewer-administered questionnaire.

### Statistical Analysis

Entry of data was done in an Access (Microsoft Corp., Red-mont, WA, USA) database. Statistical analysis was carried out using SPSS Statistics version 22.0 (IBM Corp., Armonk, NY, USA). Adherence to IPT was defined as consumption of ≥90% of doses in the preceding 2 weeks. Continuous variable, which deviated from the normal distribution, were described using median (interquartile range (IQR)). Categorical variables were summarised using frequencies and proportions. Comparisons between medians were performed using Mann-Whitney U test. Bivariate analysis and binary logistic regression analyses were used to test for the association between the dependent and the independent variables. Significance level was set at a *P*<.05.

### Ethical Considerations

Ethical clearance was obtained from KNH/University of Nairobi Ethics Committee (certificate reference number: KNH-ERC/A/234). Permission to conduct the research was sought from the relevant authorities including those of LDH, MDH and KNH. Written Informed consent was sought from the HCWs and also from the caregivers of the children who took part in the study. For confidentiality purposes, the study participants were given study identification codes and no personal identification data were recorded.

## RESULTS

The study recruited 111 child-caregiver pairs. The median (IQR) age of the caregivers was 36.0 (30.5 to 41.0) years. Most of the caregivers were female (n=77, 77.3%), married (n=60, 62.5%), HIV-positive (n=82, 85.4%) and were mothers to the children who took part in the study (67, 69.1%). Further, 28 (29.2%), 35 (36.5%), and 32 (33.3%) of the caregivers had attained, respectively, primary, secondary and college levels of education. Four caregivers (4.2%) were either on TB treatment at the time of the study or had completed the treatment within a period of not more than 6 months from the time the study was undertaken (Table 1).

**TABLE 1.** Caregiver Characteristics

Characteristic	n (%)
**Age, years (n=77)**	
<25	6 (7.8)
25–34	26 (33.8)
≥35	45 (58.4)
**Gender (n=97)**	
Male	22 (22.7)
Female	75 (77.3)
**Relationship to the child (n=97)**	
Mother	67 (69.1)
Father	19 (19.6)
Aunt	4 (4.1)
Grandmother	2 (2.1)
Sister	2 (2.1)
Other*	3 (3.1)
**Marital status (n=96)**	
Married	60 (62.5)
Widowed	7 (7.3)
Separated/divorced	5 (5.2)
Single	24 (25.0)
**Education (n=96)**	
No formal education	1 (1.0)
Primary	28 (29.2)
Secondary	35 (36.5)
College	32 (33.3)
**HIV status of caregiver (n=96)**	
Negative	14 (14.6)
Positive	82 (85.4)
**Currently on TB treatment or completed in the past 6 months (n=96)**	
Yes	4 (4.2)
No	92 (95.8)

The median (IQR) age of the enrolled children was 8.0 (9.7 to 12.6) years. Those aged less than 5 years were 9 (8.1%). The majority of children were male (n=65, 58.6%). Analysis of the household density showed that the median (IQR) density was 2.00 (1.25-3.00) persons per room. Out of 106 children (95.5%) who were on ART at the time of the study, 38 (35.8%) and sixty (56.6%) had been on ART for less than 5 years and for a period of 5 to 10 years, respectively (Table 2).

**TABLE 2.** Child Characteristics (N=111)

Characteristic	n (%)
**Age, years**	
1–4	9 (8.1)
5–9	49 (44.1)
10–12	29 (26.1)
13–15	24 (21.6)
**Sex**	
Male	65 (58.6)
Female	46 (41.4)
**Household members per room, median (IQR)**	2.00 (1.25–3.00)
**Child on antiretroviral therapy**	
Yes	106 (95.5)
No	5 (4.5)
**Years on antiretroviral therapy (n=106)**	
1 to <5	38 (35.8)
5 to 10	60 (56.6)
>10 to 15	8 (7.5)

Out of the 111 children who were eligible for IPT, 59 were found to be, either, on isoniazid at the time of the study or had received it within the 2 years preceding the survey (prevalence of 53.2% (95% confidence interval [CI] 43.9% to 62.4%). Thirty-four children (30.6%) were taking isoniazid at the time of the study while 25 children (22.5%) had been on isoniazid during the 2-year period before undertaking the study (Table 3).

**TABLE 3.** Uptake of Isoniazid Preventive Therapy (IPT) Among Participating Children (N=111)

IPT Status	n (%)
Currently on IPT	34 (30.6)
Received and completed 6 months of IPT	22 (19.8)
Received and interrupted IPT	3 (2.7)
Never received IPT	52 (46.8)
**Overall uptake (ever received IPT)**	**59 (53.2)**

Twenty-two of the 59 children (37.3%) who had a history of having been on IPT had completed the required 6 months’ treatment course. Further, 27 of the 59 children (45.8%) had not missed a dose of isoniazid during the 2 weeks before the study was initiated. Overall 28 of 59 children (47.5%) were categorised as adherent to IPT (had taken at least 90% of the prescribed doses in the preceding 2 weeks).

Education of the caregiver was partially associated with IPT uptake. Children of caregivers who had attained secondary level education were 70% less likely to having been on IPT as compared to their counterparts whose caregivers had no formal education or had reported primary school as the highest level of education achieved (odds ratio [OR] 0.30; 95% CI, 0.11 to 0.85; *P*=.021). On the other hand, having postsecond ary qualifications among the caregivers had no association with IPT uptake (*P*=.081). The rest of the attributes failed to show any statistically significant relationship with the uptake of IPT in children (Table 4).

**TABLE 4.** IPT Uptake by Caregiver Characteristics

Characteristic	Total	IPT Uptake, n (%)		OR (95% CI)	*P* Value
		Yes	No		
**Sex**					
Male	22	9 (40.9)	1 3 (59.1)	0.57 (0.22–1.51)	.256
Female	75	41 (54.7)	34 (45.3)	Reference	
**Marital status**					
Married	60	31 (51.7)	29 (48.3)	1.07 (0.47–2.44)	.874
Other	36	18 (50.0)	18 (50.0)	Reference	
**Education**					
Postsecondary	32	15 (46.9)	17 (53.1)	0.40 (0.14–1.13)	.081
Secondary	35	14 (40.0)	21 (60.0)	0.30 (0.11–0.85)	.021
None to primary	29	20 (69.0)	9 (31.0)	Reference	
**Relationship to child**					
Mother	67	37 (55.2)	30 (44.8)	2.158 (0.58–8.08)	.333
Father	19	9 (47.4)	10 (52.6)	1.58 (0.34–7.22)	.708
Other	11	4 (36.4)	7 (63.6)	Reference	
**Employment status**					
(Self-)Employed	72	37 (51.4)	35 (48.6)	1.06 (0.42–2.66)	.906
Other	24	12 (50.0)	12 (50.0)	Reference	

Abbreviations: CI, confidence interval; IPT, isoniazid preventive therapy; OR, odds ratio

A lower proportion of uptake of IPT was observed in children caregivers whose serostatus was negative when compared with those of caregivers who were seropositive. However, this association was not significant (28.6% vs 54.9% respectively, *P*=.087). Further, being on TB treatment at the time of the study or having completed the treatment showed no association with IPT uptake in children (*P*=.999). Being aware of IPT was associated with 7-fold increment in the likelihood of uptake of IPT in a child (OR 7.39; 95% CI, 2.27 to 24.08; *P*<.001). Moreover, having been on IPT among the care-givers was associated with increased probability of uptake of IPT (OR 19.50; 95% CI, 6.37 to 59.67), *P*<.001). Challenges in visiting the hospital were not associated with IPT uptake (Table 5).

**TABLE 5.** IPT Uptake by Caregiver Health-Related Factors

Factor	Total	IPT uptake, n (%)		OR (95% CI)	*P* Value
		Yes	No		
**HIV Status**					
Negative	14	4 (28.6)	10 (71.4)	0.33 (0.10–1.14)	.087
Positive	82	45 (54.9)	37 (45.1)	Reference	
**Current or completed TB treatment**					
Yes	4	2 (50.0)	2 (50.0)	0.96 (0.13–7.10)	.999
No	92	47 (51.1)	45 (48.9)	Reference	
**Aware of IPT**					
Yes	74	46 (62.2)	28 (37.8)	7.39 (2.27–24.08)	<.001
No	22	4 (18.2)	18 (81.8)	Reference	
**History of IPT use**					
Yes	40	35 (87.5)	5 (12.5)	19.50 (6.37–59.67)	<.001
No	53	14 (26.4)	39 (73.6)	Reference	

Abbreviations: CI, confidence interval; IPT, isoniazid preventive therapy; OR, odds ratio

Uptake of IPT was significantly associated with higher baseline CD4 counts (median (IQR) absolute CD4 cells/μl 790 (493–1,409.5) and 542 (309-859) in children who had ever received IPT and those who had never received IPT respectively, (z=−2.705, *P*=.007). Additionally, significantly higher current median (IQR) CD4 counts (852.0 (606.5–1,235.1)) absolute cells/μl were observed in the children who had ever received IPT as compared to their counterparts who had not received IPT (733.8 (470.4–935.2) absolute cells/μl), (z=−2. 263, *P*=.024). The age, gender of the child, frequent of clinic visits and duration on ART were not associated with IPT uptake (Table 6).

**TABLE 6.** IPT Uptake by Child Characteristics

Characteristic	Total	IPT Uptake, n (%)		OR (95% CI)	*P* Value
		Yes	No		
**Sex**					
Male	65	33 (50.8)	32 (49.2)	0.793 (0.37–1.70)	.550
Female	46	26 (56.5)	20 (43.5)	Reference	
**Frequency of clinic visits**					
<Quarterly	39	18 (46.2)	21 (53.8)	0.621 (0.28–1.37)	.237
Quarterly	69	40 (58.0)	29 (42.0)	Reference	
	**IPT Uptake, Median (IQR)**				
	**Yes**		**No**		
**Age, years, median (IQR)**	10.0 (8.0–12.0)		10.5 (8.3–13.0)		.537
**Months on ART**	72 (33–96)		60 (24–96)		.878
**Baseline CD4, absolute cells/μl**	790 (493–1409.5)		542 (309–859)		.007
**Current CD4, absolute cells/μl**	852.0 (606.5–1235.1)		733.8 (470.4–935.2)		.024

Abbreviations: CI, confidence interval; IPT, isoniazid preventive therapy; IQR, interquartile range; OR, odds ratio

The association between caregivers’ characteristics and uptake of IPT was evaluated using binary logistic regression and the outputs are presented in Table 7. Age of the caregiver, though not associated with IPT uptake after univariate analysis, was included in the multivariate analysis due its potential confounding effect. Overall, education was found to be associated with uptake of IPT (*P*=.047). In particular, children whose caregivers had attained secondary school level of education were found to be 87% less likely to have received IPT when compared with those of caregivers who had no formal education or had achieved Primary school as the highest level of education (adjusted odds ratio [aOR] 0.13; 95% CI, 0.03 to 0.67; *P*=.014). Additionally, children whose caregivers had a history of being on IPT had an increased likelihood of having received IPT (aOR 27.50; 95% CI, 5.39 to 140.28; *P*<.001). Analysis of the ages of caregivers showed that a unit increase in age resulted in about 8% increment in the probability of IPT uptake in children. However, this relationship failed to attain statistical significance (aOR 1.08; 95% CI, 0.99 to 1.17; *P*=.088).

**TABLE 7.** Multivariate Analysis Outputs

Variable	aOR (95% CI)	*P* Value
**Age, years**	1.08 (0.99–1.17)	.088
**Education**		.047
None to primary	Reference	
Postsecondary	0.28 (0.06–1.39)	.119
Secondary	0.13 (0.03–0.67)	.014
**Aware of IPT**		
Yes	Reference	
No	1.68 (0.33–8.46)	.531
**History of IPT**		
Yes	Reference	
No	27.50 (5.39–140.28)	<.001

Abbreviations: aOR, adjusted odds ratio; CI, confidence interval; IPT, isoniazid preventive therapy

To assess knowledge, attitude and practices of HCWs with regard to provision of IPT, we interviewed 66 HCWs, most of whom were females and working in the Paediatric Inpatient Department (43 HCWs [65.2%] in both cases). Assessment of the HCWs’ knowledge showed that, respectively, 59 (89.4%) and 2 (3.0%) HCWs knew that isoniazid and BCG vaccine could be used to prevent TB. Further, 47 HCWs (71.2%) stated the correct IPT dosage (10 mg/kg/day). Additionally, 59 HCWs (89.4%) correctly reported that the drug is administered once daily, while 50 HCWs (75.8%) reported the correct duration for the IPT course (6 months).

A total of 61 HCWs (92.4%) responded on the affirmative on being asked if TB is preventable. Additionally, the majority of HCWs (n=64, 97.0%) rated IPT as either effective or very effective. Those who reported that they were comfortable or very comfortable with administering IPT were 53 (80.3%). Half of the HCWs (n=33, 50.0%) rated the side effects of IPT as mild or very mild. Ease of following IPT guidelines was rated as either easy or very easy by 56 HCWs (84.8%). Overall, 54 HCWs (81.8%) were considered to have a favourable attitude towards IPT.

Analysis of the practices revealed that 45 HCWs (68.2%) had ever started a patient on IPT. In addition, 33 HCWs (50%) reported that they had started at least 1 patient on IPT in the 12 months preceding the survey. Twenty-four HCWs (36.4%) had ever renewed an IPT prescription, while 21 HCWs (31.8%) stated that they had prescribed IPT in the 1-year period before the study.

## DISCUSSION

Our study demonstrated poor uptake of IPT among children living with HIV/AIDS with only about half of the eligible children having been initiated on IPT. The uptake documented in the current study is not very different from that of a research done in the Eastern Cape Province of South Africa where the uptake of IPT among children aged not more than 15 years was 58.7%.^23^ A research done in Riruta Health Centre, Nairobi, Kenya, recorded a higher uptake of IPT (77%).^24^ The higher IPT uptake could be due to the fact that the study recruited adults (≥18 years) rather than children as is the case in our study. Additionally, as compared to our study, a slightly higher IPT initiation rate was reported in South Africa and Ethiopia where initiation of IPT was done in 68%^25^ and 64%^26^ of the children who were eligible for IPT, respectively. On the other hand, the uptake of IPT in our study was higher than the 37% reported in Ethiopia,^27^ 33% rate in southern India,^28^ the 18% demonstrated in Timor-Leste,^29^ the 6% reported in a Malawian study^30^ and 22% in Bhopal, India.^31^ The high variations in the findings most probably is a reflection of the contextual differences in the settings where the studies were carried out.

The present study demonstrated good completion of IPT once initiated with about 4 out of 5 children started on IPT completing the 6 month treatment course. The low non-completion rate (about 1 in every 5 children) could partly be explained by the high percentage (95.5%) of participants concomitantly being on antiretroviral therapy. As a result, patients had an incentive to come to the clinic already that was independent of IPT thus removing any additional travel and time burden related to acquiring IPT medication. This finding reiterates the observations made by a Malaysian study whereby patients receiving concurrent highly active antiretroviral therapy had significantly higher IPT completion rate when compared to their counterparts who were not on HAART.^32^ In concordance with our findings, high IPT completion rates were documented in Ethiopia, Malaysia and Democratic Republic of Congo where, respectively 80%,^26^ 81%^32^ and 88%^33^ of the patients completed the 6-month IPT course. Contrary to our findings, some studies have shown lower IPT completion rates: Ethiopia (67.9%),^27^ India (20%)^31^ and South Africa (3.7%).^23^

The reasons for noncompletion were as a result of poor patient compliance and high pill burden. In India, stock-outs of isoniazid, long treatment duration and side effects were stated as the reasons for noncompletion of IPT.^28^ Studies have shown that compliance with shorter courses of TB preventive therapy, that is, 3 or 4 month courses of isoniazid and rifampicin or rifampicin alone, is significantly better.^34–37^ Moreover, the drugs used in the shorter courses are available in child friendly formulations.^37^ Adoption of the shorter treatment courses in the current treatment guidelines should be thus be considered.

Adherence is a principal determinant of efficacy of IPT.^38^ In the resent survey adherence was good; about 4 in 5 children never missed taking isoniazid for the entire period under consideration. The proportion of the participants who were categorised as adherent (had taken at least 90% of the prescribed doses in the preceding 2 weeks) was 82.4%. The level of adherence in our study was not very different from what was reported in Ethiopia (86.1%)^27^ and Uganda (80.0%).^39^ On the contrary, a study done in Gambia, adherence was 61% which could probably be explained by the fact that the study involved home-based IPT delivery.^40^ Furthermore, the Gambian study relied on pill count, rather than self-reports, to assess the adherence. Self-reports are prone to over-estimation of the phenomenon under research. The observed high rates of adherence and completion rates could be an indication that IPT is highly acceptable to the children and their care givers. It could also suggesting that system-related rather than client-related factors are the chief determinants of successful implementation of IPT.

An inverse trend in the uptake of IPT was noted as care-giver's educational attainment increased. Contrary to what our data indicated, generally it is expected that uptake of health services improves with increasing education attainment. The reason for this anomalous finding is not clear and, perhaps calls for further investigation. We hypothesise that could a reflection of the differences in perception of vulnerability to TB among people with different levels of education. Nonetheless, some previous studies have indicated heterogeneity in utilisation of health services based on education attainment particularly preventive health services. For instance, a research led by Kim found that people with higher education were less likely to get vaccinated, but more likely to utilise other preventive services such as cancer screening.^41^ Moreover, research by Labeit and colleagues revealed that the positive influence of higher education on uptake of recommended medical check-ups in the UK was only visible in dental screening and not on cancer screening (breast and cancer), blood pressure checks and cholesterol tests.^42^ Contrary to our findings, education was not a significant predictor of IPT uptake in a study conducted in India.^31^ However, the Indian study was only interested in the mother's literacy rather than the caregiver's education which could be a possible reason for the discordant results.

Children whose caregivers had a history of being on IPT were more likely to have received IPT. The finding reiterates the findings of a study by Skinner et al^43^ who revealed that caregivers’ refusal to medicate was a significant barrier to providing children with IPT.

In addition, the child's CD4 count (both baseline and current) were significant predictors of uptake of IPT. Most probably this finding is a reflection of the eligibility criteria for initiation of ART (such as CD4 ≤500 cells/mm^3^) at the time the study was undertaken).^44^

Studies done in other places have documented correlates of IPT uptake which were not found to be significant determinants of IPT uptake in the current study. For instance, in South India, social stigma was the key barrier of IPT uptake.^45^ A community-based survey carried out in Malawi demonstrated that transport cost for getting chest radiography done was the main reason for low uptake of IPT.^46^ A study by Lester et al^47^ documented lack of knowledge on IPT as a key barrier. Research done in Timor-Leste reported difficult terrain as main barrier for IPT uptake.^29^

These findings are concordant with those of a study done in South Africa which cited lack of experience, knowledge, and clarity on the benefits of IPT and existing guidelines by HCWs as important barriers for IPT provision rather than patient-related factors.^48^ Similarly, inadequate knowledge and training of health-care providers and poor monitoring mechanism in the programme were noted as key bottlenecks in the provision of IPT in India.^49^ In Kigali, Rwanda, unfriendly health-care providers were highlighted as a key barrier in utilisation of IPT.^50^ Further, research done in Lesotho noted that inadequate HCW education on IPT implementation was negatively impacting on the implementation of IPT in Lesotho.^51^

Generally, the current survey revealed that nearly nine out ten HCW had a favourable attitude with regard to IPT, its administration and the user-friendliness of the guideline. Discordant with this finding, another study conducted in the same setting highlighted that healthcare providers (HCP) expressed discomfort with the IPT guidelines and standard operating procedures (SOPs) citing that the documents lacked clarity. In particular, the HCP reported that the guidelines on eligibility criteria, on how to decide whether a patient had active and latent TB and on the duration of IPT were unclear.^52^ The fact that the later study sampled both clinical and non-clinical HCP while our research recruited HCWs who were working in areas where IPT was being provided could most probably explain the differences observed in the findings between the two studies.

It is noteworthy that, in the present study only one-half of the HCW perceived the side effects associated with use of isoniazid as mild or very mild. It is possible that such a negative perception of the drug's safety and tolerability may make the HCW to be reluctant in prescribing it. Contrary to our study findings, an Indian study documented a lower proportion of HCW who had had a favourable attitude: only 56% of the HCW believed IPT would benefit children by preventing TB.^49^

Our study showed that prescription is a potential hindrance in the uptake of IPT given that only one half of the HCW reported that they had started at least one patient on IPT in the twelve months preceding the survey. Furthermore, only about a third of the HCW had ever renewed an isoniazid prescription. Given the high proportion of HCW who were knowledgeable and also had a favourable attitude towards IPT, the observed reluctance of staff to prescribe IPT may be due to the fear of side effects associated with IPT. It could also be due to challenges in implementing the screening process such as the requirement to rule out active TB before starting IPT. In line with this, a study showed that the belief that existing screening tools are inaccurate in HIV-infected individuals and the need to refer patients to separate clinics for tuberculosis screening has been shown to be barriers in utilization.^48^ Besides, the attendant lack of experience in administering IPT among HCW may also be a contributor to the low rates of prescription of IPT. One strategy to counter this could be use of ‘IPT champions’ who would set an example by prescribing IPT thus promoting other HCW to follow suit.^52^ Additionally, supervision with provision of feedback concerning IPT delivery might also improve implementation.^53^

### Limitations

Our research study is not without limitations. The determination of the sample size of the study utilized a relative lower level of precision (10%) compared to most studies which use the conventional 5% as the desired precision. The resultant sample size was small hence wider estimates of confidence intervals for the study variables. It is also possible that the study could have missed some significant associations between variables due to the low statistical power associated with lower sample sizes. Further, the findings from our study may not be generalisable to other counties in Kenya considering that the study was based in an urban setting. The results may also not be generalisable to the entire city of Nairobi since KNH, being a referral hospital, gets patients from all corners of the country. The participants were enrolled from in a single hospital and thus could have missed to capture the diversity of the population. The facility was a referral hospital and thus the findings may have limited generalizability for other public health facilities. Nevertheless, the findings in the current provides evidence about the implementation of IPT in the programmatic settings and, thus, potentially reflects the realities on the ground.

## CONCLUSION

The uptake of IPT demonstrated in the current study was sub-optimal. This represents a missed opportunity to prevent future TB cases. On the other hand, the study demonstrated good IPT completion rates and adherence. This is encouraging, but more effort is needed to ensure 100% completion and adherence. Achievement of a higher IPT completion rate in this study also demonstrates that IPT is feasible in a resource-limited high TB-burden setting.

The gaps in HCW are likely to have resulted in delayed or lost opportunities for prevention hence the low uptake of IPT. There is, thus, a need to be address the gaps. This may be achieved by training or retraining HCWs on TB/IPT, regular on-the-job sensitization about IPT and perhaps selecting HCW who could champion the implementation of IPT including prescribing isoniazid.
